# Mitogen-Activated Protein Kinases: Therapeutic Signaling Catalysts in Viral Immune Evasion

**DOI:** 10.3390/pathogens15040384

**Published:** 2026-04-03

**Authors:** Masood Alam Khan, Mohammad Hamza Khan, Khaled S. Allemailem

**Affiliations:** 1 Department of Basic Health Sciences, College of Applied Medical Sciences, Qassim University, Buraydah 51452, Saudi Arabia; 2 Jawaharlal Nehru Medical College and Hospital, Aligarh Muslim University, Aligarh 202002, India; 3 Department of Medical Laboratories, College of Applied Medical Sciences, Qassim University, Buraydah 51452, Saudi Arabia

**Keywords:** MAP kinase, viral protein, virus pathogenesis, immunity, vaccination

## Abstract

The mitogen-activated protein kinase (MAPK) pathways, ERK, JNK, and p38, are key regulators of immune responses during viral infections. These signaling cascades control cytokine production, T cell activity, and antigen presentation. However, many viruses can hijack MAPK pathways to avoid immune detection, promote their replication, and establish chronic infection. In this review, we discuss how different viruses, including HSV-1, HBV, HCMV, and SARS-CoV-2, manipulate MAPK signaling to alter host cell functions. A particular focus is given to the CD1d–iNKT cell axis, which plays a critical role in early antiviral responses but is often disrupted through MAPK-dependent mechanisms. We explore how changes in MAPK signaling affect antigen-presenting cells, drive T cell exhaustion, and reprogram immune cell metabolism, factors that contribute to viral immune evasion. The review also examines therapeutic strategies aimed at targeting MAPKs to improve antiviral immunity. These include small-molecule inhibitors and immune modulators that may enhance antiviral responses while limiting side effects. We emphasize the importance of context, as MAPK-targeted therapies must be carefully timed and tailored to avoid suppressing protective immunity or triggering unwanted inflammation. Overall, this review highlights the therapeutic potential and challenges of targeting MAPK pathways in viral infections and encourages further research into selective, host-directed antiviral strategies.

## 1. Introduction

Viruses and the host immune system are locked in a constant evolutionary struggle, with intracellular signaling pathways serving as key battlegrounds. Among these, the mitogen-activated protein kinase (MAPK) cascade, including the ERK1/2, p38, and JNK subfamilies, plays a central role in regulating immune responses, cell survival, apoptosis, and antigen presentation [1,2,3,4,5,6]. Rather than being passive responders to infection, these kinases are often actively manipulated by viruses to promote replication, suppress immune recognition, or reprogram host cell behaviour.

MAPK activation during viral infection is not uniform. It varies across cell types, infection stages, and viral species, producing both protective and pathological effects [7,8,9,10]. For example, SARS-CoV-2 activates p38 signaling to drive pro-inflammatory cytokine production, contributing to lung injury. Yet, the same pathway is also essential for inducing interferon-stimulated genes and maintaining epithelial barrier integrity [11,12,13]. This illustrates the dual role of MAPKs: necessary for antiviral defense but potentially harmful when dysregulated. However, much of the existing literature treats these pathways as linear and unidirectional, overlooking the complexity of compensatory signaling, pathway cross-talk (e.g., with PI3K/Akt, NF-κB, JAK/STAT), and temporal regulation. Conflicting findings further complicate the narrative. Herpes simplex virus (HSV-1), human immunodeficiency virus (HIV-1), and hepatitis B virus (HBV) have each been reported to both activate and inhibit MAPK signaling, depending on the infection context [14,15,16,17,18]. Such variations are not inconsistencies but reflect the flexible nature of host–virus interactions. From a therapeutic standpoint, this variability has significant implications. p38 inhibitors, while effective in controlling inflammation, may impair early antiviral signaling [12,19], and ERK modulation, though promising, carries risks due to its involvement in cell proliferation and tumorigenesis [20].

A particularly underexplored dimension of MAPK involvement in viral immunity is its regulation of non-classical antigen presentation, especially through the CD1d-invariant natural killer T (iNKT) cell axis. Unlike classical MHC molecules, CD1d presents lipid antigens to iNKT cells, initiating rapid cytokine responses and bridging innate and adaptive immunity [21,22,23]. Multiple viruses, including vaccinia virus, HSV-1, and vesicular stomatitis virus (VSV), interfere with CD1d expression or trafficking to evade iNKT-mediated surveillance, often by hijacking MAPK signaling components [24,25,26,27,28,29]. Despite its immunological importance, the mechanistic links between MAPKs and CD1d regulation remain poorly defined. The roles of upstream lipid metabolism, ER stress pathways, and ubiquitination are emerging but not fully understood.

This review aims to critically synthesize current knowledge on how viruses exploit MAPK pathways to modulate immune responses, particularly by interfering with iNKT cell activation and CD1d presentation. Instead of reiterating canonical MAPK pathway functions, we focus on areas of contradiction, emerging hypotheses, and overlooked regulatory layers. By highlighting virus-specific strategies and the interplay between signaling and antigen presentation, we outline a roadmap for future therapeutic targeting that balances antiviral efficacy with immune homeostasis.

## 2. Beyond the Canon: Dynamic Roles of MAPK Subfamilies in Viral Infections

**Figure 1:**
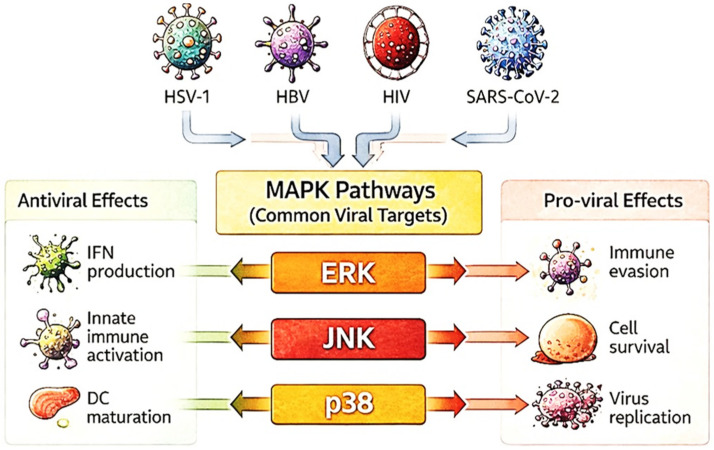
Diverse viruses, including HSV-1, HBV, HIV, and SARS-CoV-2, target MAPK pathways (ERK, JNK, and p38) to influence host immune responses. These pathways mediate both antiviral functions, such as interferon (IFN) production, DC maturation, and innate immune activation, and pro-viral effects, including immune evasion, enhanced cell survival, and viral replication.

The MAPK family, composed of ERK1/2, p38, and JNK kinases, operates far beyond its classical roles in proliferation and stress response, playing critical and multifaceted roles during viral infection (Figure 1). During viral infections, each subfamily functions within a tightly regulated, context-specific network, sometimes supporting host defense, other times enabling viral persistence. This complexity challenges simplified interpretations and requires a deeper examination of how MAPKs contribute to both protective immunity and immune subversion.

ERK1/2, traditionally linked to cell growth and proliferation, demonstrates opposing roles in infection. In T and B cells, ERK activation promotes proliferation, cytokine production, and differentiation, key components of effective antiviral immunity [30,31,32]. However, viruses such as human cytomegalovirus (HCMV) and HSV-1 exploit ERK signaling in infected epithelial or myeloid cells to prevent apoptosis, support latency, or enhance viral replication [12,19,33]. This cell-type specificity highlights ERK’s dual nature: beneficial in immune cells, yet exploitable in permissive cells. Consequently, therapeutic ERK inhibition must be approached cautiously, as systemic blockade may impair essential immune functions [8,18].

The p38 MAPK pathway, activated in response to stress and inflammation, governs transcription of pro-inflammatory cytokines, DC maturation, and macrophage polarization [9,34,35]. It is robustly induced in infections such as SARS-CoV-2, influenza, and HBV, where it contributes to cytokine storms and tissue damage [4,11,12]. At the same time, p38 is necessary for type I interferon (IFN) signaling, making it a paradoxical therapeutic target: inhibition may reduce inflammation but also compromise antiviral defense [11,35]. Moreover, certain viruses selectively activate p38 to suppress antigen presentation pathways, including CD1d expression, as observed in vaccinia virus and VSV infections [7,23,24].

Though less extensively characterized, JNK (c-Jun N-terminal kinase) also plays a pivotal role in host–pathogen interactions. It regulates apoptosis, autophagy, and differentiation of immune cells [5,6,7]. Viruses like hepatitis C virus (HCV) and HIV-1 manipulate JNK signaling to alter T cell survival and impair antigen-presenting cell function, prolonging infection and weakening host immunity [16,17]. Additionally, JNK influences DC activation and T helper (Th1/Th2) polarization, suggesting a broader immunomodulatory role during infection [7,9].

Crucially, MAPK subfamilies do not operate in isolation. Crosstalk and feedback mechanisms between ERK, p38, and JNK pathways enable flexible responses to viral cues. Inhibiting one arm, for example, p38, can enhance signaling through ERK, with divergent effects ranging from immune tolerance to hyperproliferation [12,19]. Viruses often exploit this by dampening specific MAPKs while preserving others, fine-tuning the intracellular environment to their advantage. Understanding this compensatory landscape is essential for designing targeted therapies that can modulate MAPK activity without triggering unintended immune or oncogenic consequences. To further highlight how viral modulation diverges from canonical host MAPK signaling in terms of activation dynamics, spatial organization, and downstream immune outcomes, a comparative summary is provided in Supplementary Table S1.

## 3. Viral Modulation of MAPK Signaling: Precision Hijacking or Contextual Exploitation?

Importantly, viral manipulation of MAPK signaling occurs within a highly interconnected network, where ERK, JNK, and p38 exhibit compensatory and cooperative dynamics; thus, modulation of one pathway often reshapes the activity of others, collectively determining infection outcomes. Within this networked framework, viruses exploit MAPK pathways not as simple binary switches but as complex, context-dependent signaling hubs. Viruses such as HSV-1, HBV, HIV, and SARS-CoV-2 commonly target ERK, JNK, and p38 pathways to modulate host immune responses. Depending on the viral strategy, stage of infection, and host cell type, MAPK activation can drive either antiviral outcomes, such as interferon production and dendritic cell maturation, or pro-viral effects, including immune evasion, enhanced replication, and cell survival (Figure 1). Rather than uniformly activating or suppressing these cascades, many viruses finely tune MAPK activity in a temporal and cell-type-specific manner, enabling dynamic adaptation to the host environment. This precise regulation allows viruses to optimize replication conditions while minimizing immune detection and promoting persistence. Understanding these virus-specific and network-driven strategies is essential for disentangling the nuanced roles of MAPKs in immunity and viral pathogenesis.

One common tactic employed by viruses is early and transient activation of MAPKs during the initial stages of infection. This brief signaling burst facilitates crucial viral processes such as nuclear import, cytoskeletal rearrangement, and initiation of viral gene transcription, before the host’s antiviral defenses are fully mobilized. For example, influenza A virus, HBV, and human cytomegalovirus (HCMV) are known to activate MAPKs such as ERK and p38 shortly after entry [15,36,37]. In the case of HCMV, ERK activation supports the survival of infected myeloid cells during latency by inducing anti-apoptotic proteins like MCL-1, helping the virus persist in immune-privileged reservoirs [32,38].

In contrast, other viruses employ selective suppression of MAPK pathways to blunt immune responses. The HSV-1 protein ICP0 degrades upstream MAPK signaling adaptors, indirectly dampening stress kinase activation and delaying immune detection [14]. Similarly, during chronic stages of infection, HIV-1 downregulates JNK and p38 signaling in myeloid DCs, weakening their capacity to present antigens and produce cytokines [16]. SARS-CoV-2 takes this suppression further by using accessory proteins like ORF6 and ORF7a to interfere with downstream components of p38 and JNK pathways, preventing activation of interferon-stimulated genes crucial for antiviral defense [11,39].

Importantly, the same virus may modulate different MAPK pathways depending on the host cell type. This divergent modulation allows viruses to exploit tissue-specific vulnerabilities. For example, HIV-1 enhances ERK signaling in T cells to support viral integration and survival, but suppresses p38 activation in dendritic cells to disrupt immune priming [16,40]. Likewise, SARS-CoV-2 induces robust p38-mediated cytokine responses in epithelial cells, contributing to inflammation and lung pathology, while exerting minimal MAPK activation in monocytes, where interferon signaling predominates [11,41]. These observations underscore the strategic flexibility of viruses in hijacking MAPK pathways to their advantage, shaped by both viral and host–cell determinants.

Apparent inconsistencies in viral modulation of MAPK signaling largely reflect contextual and methodological variability rather than true biological conflict. MAPK responses differ across cell types, with epithelial cells, T cells, and antigen-presenting cells exhibiting distinct baseline signaling and immune functions [12,34]. Outcomes are further shaped by infection parameters, including multiplicity of infection, viral strain, and stage of infection, which dictate whether MAPK activation is transient, sustained, or selectively biased [15,36,37]. Discrepancies also arise from divergent analytical endpoints, as studies assess MAPK activity at different levels, phosphorylation, transcriptional outputs, or functional immune responses, capturing non-equivalent aspects of pathway behavior [9,20]. Critically, temporal resolution remains underappreciated, despite evidence that MAPK signaling is dynamically reprogrammed throughout infection [11]. Together, these variables underscore that viral manipulation of MAPKs is inherently context-dependent, and that seemingly contradictory findings often reflect differences in experimental design rather than opposing mechanisms.

## 4. Viral Signatures in MAPK Hijacking: Comparative Insights Across Pathogens

Although many viruses target the MAPK signaling cascade, they do so in virus-specific ways that reflect differences in their replication strategies, tissue tropism, and interactions with the immune system. Rather than acting through a single shared mechanism, each virus selectively manipulates components of the MAPK network to achieve immune evasion, enhance replication, or establish latency (Table 1). This section highlights the diverse strategies used by major viral families, offering a comparative view of how MAPK hijacking is tailored to viral context (Table 1).

Herpesviruses, including Epstein–Barr virus (EBV), HSV-1, and HCMV, exemplify dual-use of MAPK signaling for both latency maintenance and reactivation. EBV sustains low-level ERK activity through its latent membrane protein LMP1, supporting B cell survival and promoting oncogenic transformation. Upon reactivation, it upregulates MAPKs through immediate-early proteins BZLF1 and BRLF1 to initiate lytic gene expression [25,33,42]. HSV-1 manipulates JNK and p38 through its tegument proteins and ICP0, suppressing early immune responses in antigen-presenting cells while later activating ERK to drive viral DNA replication [14,26]. Similarly, HCMV co-opts both ERK and p38 in myeloid progenitor cells to maintain latent reservoirs and promote cell survival [32,38].

In SARS-CoV-2 infection, MAPK signaling plays a double role. Activation of the p38 pathway contributes to lung inflammation, neutrophil recruitment, and the cytokine storm that drives severe disease. However, p38 is also necessary for effective type I interferon (IFN) responses and maintaining epithelial barrier integrity [11,13,41]. Viral proteins such as the nucleocapsid (N) protein and ORF7a interact with MAPKs to either promote or suppress their activation depending on the infection stage and host cell type [40,43]. This dual function makes therapeutic targeting of MAPKs in COVID-19 both promising and complex.

Hepatitis viruses, particularly HBV and HCV, exploit MAPK pathways to support chronic infection and liver disease progression. HBV activates ERK to promote hepatocyte proliferation, viral gene expression, and immune evasion without triggering strong inflammation [17,44]. HCV, through its envelope protein E2, stimulates ERK and JNK, promoting anti-apoptotic signaling and skewing cytokine responses, mechanisms linked to liver fibrosis and hepatocellular carcinoma [45]. These viruses manipulate MAPKs not only for survival but also to reshape cellular metabolism and antigen presentation in the liver.

In influenza A virus infection, MAPKs serve as temporary replication supports rather than tools for long-term immune escape. ERK activation is crucial for the nuclear export of viral ribonucleoprotein complexes (vRNPs), a key step in virus assembly [24,46]. Meanwhile, p38 and JNK contribute to stress responses and facilitate viral replication in respiratory epithelial cells, though they also help recruit immune cells [4,47,48]. Targeting these pathways has shown potential to reduce viral replication while preserving host immunity, positioning influenza as a model for MAPK-based antiviral strategies [12,13].

HIV-1 displays highly compartmentalized MAPK modulation. In CD4+ T cells, the virus activates ERK to promote integration of viral DNA and enhance host cell survival [16]. In contrast, in DCs and macrophages, HIV suppresses p38 and JNK signaling to impair antigen presentation and reduce expression of costimulatory molecules, helping the virus avoid immune detection [16,40]. This cell-type specificity highlights the virus’s ability to tailor MAPK modulation to the functional role of the infected cell. Together, these pathogen-specific examples illustrate that MAPK signaling is not manipulated uniformly across viral infections. Instead, viruses fine-tune their interference based on cellular context and disease stage, reinforcing the need for precision approaches when considering MAPKs as antiviral targets.

**Table 1:** Proteins of Viruses Targeting MAPK Pathways: Mechanisms of Modulation and Immune Evasion.

Virus	Viral Protein/Factor	MAPK Pathway Affected	Mechanism of Modulation	References
HSV-1	ICP0	p38, JNK	ICP0 indirectly targets MAPKs via ubiquitin-mediated mechanisms	[14,26]
HCMV	UL97, IE86	ERK, p38	UL97 activates ERK; IE86 upregulates MCL-1 through ERK	[32,38,49]
HBV	HBx	ERK, JNK	HBx activates ERK and JNK; modulates cell cycle and transformation	[17]
HCV	E2 protein	ERK, p38, JNK	E2 activates MAPKs, leading to enhanced viral entry and replication	[44,45]
EBV	LMP1, BZLF1	ERK, p38	LMP1 activates ERK/p38 for transformation; BZLF1 triggers lytic cycle via MAPK	[25,33,42,50]
Vaccinia Virus	VH1, B1R, H5R	ERK, JNK, p38	VH1 dephosphorylates MAPKs; B1R/H5R inhibit CD1d antigen presentation	[28,51,52]
SARS-CoV-2	ORF3a, ORF7a, NSP1	p38, ERK	ORFs modulate MAPK pathways for immune evasion and inflammation	[12,39,53]
VZV	Unspecified	p38 (implicated)	Downregulation of CD1d and antigen presentation (MAPK role speculative)	[27]
HIV-1	Tat	ERK, JNK, p38	Tat activates ERK/p38; contributes to T cell dysfunction and apoptosis	[16]

## 5. MAPK Signaling in Immune Dysregulation: Beyond Cytokine Storms

**Figure 2:**
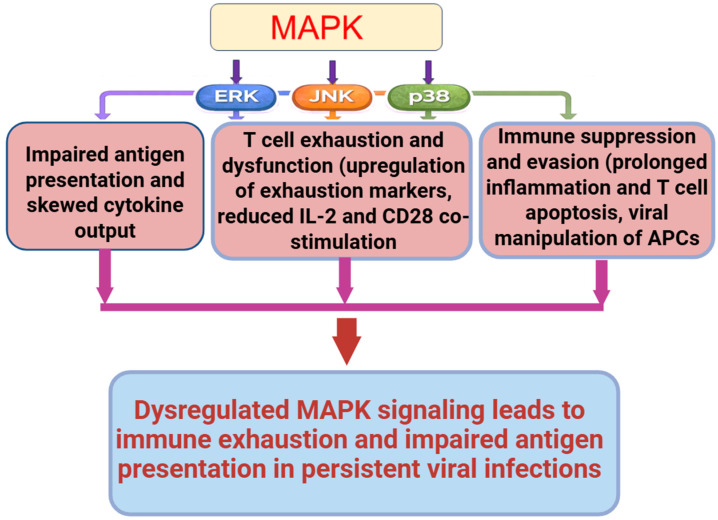
Multifaceted Roles of MAPK Pathways in Immune Dysregulation during Viral Infections. MAPK signaling pathways (ERK, JNK, and p38) coordinate multiple layers of immune regulation beyond inflammation. MAPKs influence antigen presentation and T-cell programming, cytokine balance, immune cell metabolism, and antiviral defense. Early during infection, transient ERK and p38 activation supports pro-inflammatory cytokine production and initial immune defense, whereas sustained or dysregulated MAPK activity in chronic viral infections drives immune exhaustion, impaired antigen presentation, metabolic dysfunction, and immune suppression. The biphasic trajectory highlights the importance of timing and duration of MAPK signaling in shaping protective versus pathological immune outcomes.

MAPK pathways do more than amplify inflammation during viral infections; they also coordinate multiple layers of immune regulation (Figure 2). From antigen presentation and T cell programming to cytokine balance and cellular metabolism, MAPKs play both protective and pathological roles. When dysregulated, they contribute to immune exhaustion, impaired antigen presentation, and metabolic dysfunction, especially in chronic viral infections. This section explores these underappreciated dimensions of MAPK involvement in immune dysregulation.

MAPK signaling during infection is not static; it often follows a biphasic trajectory (Figure 2). In the early phase, viruses like SARS-CoV-2 and HIV trigger rapid activation of ERK and p38 in epithelial and immune cells, leading to pro-inflammatory cytokine release, including IL-6, TNF-α, and IFN-β [4,16]. This response aids initial defense but can be overzealous. In later stages, sustained or unresolved MAPK activity contributes to immune suppression. For instance, chronic ERK activation in HIV enhances early viral replication but later drives T cell exhaustion and weakens memory formation [16,54,55]. Similarly, prolonged p38 activity in SARS-CoV-2 has been associated with lymphopenia and dysfunctional T cells, even after the virus is cleared [47,54,56,57]. These findings suggest that the timing and duration of MAPK signaling critically shape infection outcomes.

MAPKs direct key decisions in T cell differentiation and longevity. ERK promotes effector T cell expansion and cytokine secretion, yet its overactivation can drive terminal differentiation at the cost of forming long-lived memory cells [29,43]. JNK regulates T cell apoptosis, especially during overstimulation, thus shaping immune contraction phases. Meanwhile, p38 is increasingly linked to T cell senescence and loss of responsiveness, particularly in chronic infections or aging contexts. In diseases such as HIV, HBV, and HCV, prolonged MAPK signaling contributes to the upregulation of exhaustion markers like PD-1, weakening T cell function [54]. This is compounded by reduced IL-2 production and disrupted CD28 co-stimulation [16], leading to a collapse in polyfunctional T cell responses necessary for virus control [43].

MAPKs also regulate the function of DCs and macrophages, key antigen-presenting cells (APCs) responsible for initiating T cell responses. These pathways influence cytokine profiles that guide T helper cell polarization (e.g., Th1, Th2, Th17), as well as the expression of co-stimulatory molecules like CD80 and CD86, and the trafficking of MHC molecules. Viruses such as EBV, HIV, and SARS-CoV-2 subvert these functions by altering MAPK activity to skew cytokine output toward immunosuppressive IL-10 and TGF-β [11,25,43], while also reducing the surface expression of MHC-II and co-stimulatory markers [43,58,59]. This impairs T cell priming and enables persistent viral infection through immune evasion.

Beyond signaling, MAPKs regulate the metabolic programs that fuel immune cell function. ERK and p38, in particular, influence glucose metabolism and mitochondrial respiration, important processes for sustaining antiviral responses. In chronic infections like HIV and HBV, dysregulated MAPK signaling alters metabolic checkpoints, leading to immune cell exhaustion and viral persistence [16,17,43]. Such metabolic rewiring reduces ATP production and biosynthesis, impairing T cell activation and survival. These effects may not be evident from cytokine profiles alone but are crucial for immune competence. Therefore, targeting MAPKs therapeutically must consider their metabolic impact to avoid unintentionally weakening immune energy reserves.

## 6. MAPK Regulation of the CD1d–iNKT Axis: A Strategic Checkpoint in Viral Immune Evasion

**Figure 3:**
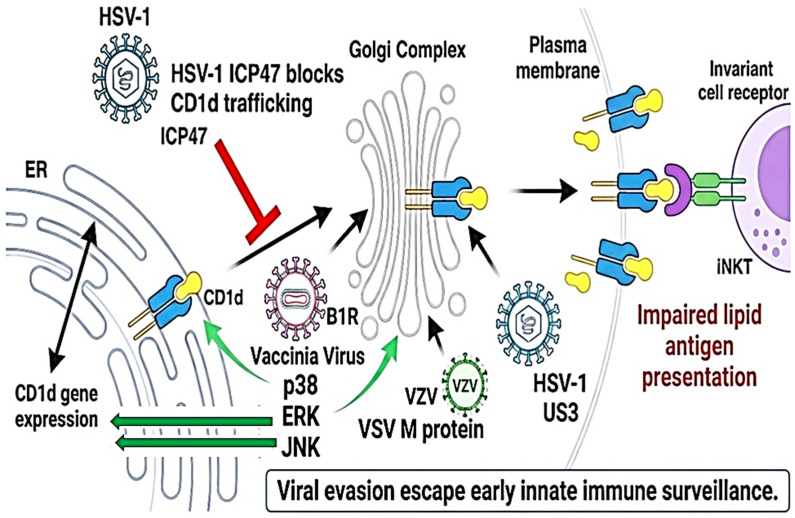
Viruses disrupt CD1d-mediated lipid antigen presentation by interfering with intracellular trafficking and MAPK signaling (ERK, JNK, p38). Viral proteins such as HSV-1 ICP47, vaccinia B1R, and VSV M protein reduce CD1d surface expression, impair iNKT cell activation, and enable escape from early innate immune surveillance.

The CD1d–iNKT cell axis is a critical part of the antiviral immune response, acting at the crossroads of innate and adaptive immunity. Unlike classical MHC molecules, CD1d presents lipid and glycolipid antigens to iNKT cells, leading to rapid cytokine release, cytotoxic responses, and activation of downstream immune players such as DCs and NK cells (Figure 3) [22]. Because of its speed and potency, this pathway is a prime target for viral interference. Many viruses, including HSV-1, varicella zoster virus (VZV), vaccinia virus, and HCMV, have developed strategies to suppress CD1d surface expression or disrupt its intracellular trafficking, allowing them to escape early immune detection by iNKT cells (Table 2, Figure 3) [16,28,60].

Emerging research points to the MAPK signaling network, particularly the p38 and ERK subfamilies, as central regulators of CD1d dynamics. These kinases influence multiple steps in CD1d processing, including biosynthesis, retention in the endoplasmic reticulum (ER), and recycling to the cell membrane (Figure 3) [61,62]. For example, viral infection-induced p38 activation is associated with CD1d accumulation inside the cell, implying a trafficking blockade rather than a transcriptional shut-off. On the other hand, moderate ERK activity has been linked to improved CD1d surface expression and antigen-loading efficiency. Viral proteins such as vaccinia virus B1R and H5R seem to hijack these pathways, activating MAPK intermediates that interfere with CD1d transport, ultimately reducing its accessibility to iNKT cells (Table 2, Figure 3) [28]. However, the exact molecular mechanisms, whether through ER chaperones, adaptor proteins, or vesicular trafficking regulators, remain insufficiently characterized. To move beyond descriptive associations, a more mechanistic framework is needed to define how MAPK signaling regulates CD1d trafficking and function. We propose that MAPKs influence the CD1d pathway through at least three interconnected regulatory nodes. First, MAPKs may modulate endoplasmic reticulum (ER) quality control and chaperone systems, including calreticulin-dependent folding and assembly, thereby affecting CD1d maturation and export. Second, MAPK signaling may regulate endosomal recycling and vesicular trafficking, potentially through control of Rab GTPases, cytoskeletal dynamics, or adaptor complexes that determine CD1d routing between endolysosomal compartments and the plasma membrane. Third, MAPKs may influence post-translational modifications of CD1d or associated trafficking proteins, such as phosphorylation or ubiquitination, which could alter intracellular retention, recycling efficiency, or degradation. These hypotheses are testable using integrated experimental approaches, including selective genetic or pharmacological perturbation of ERK, p38, and JNK pathways combined with quantitative CD1d trafficking assays, live-cell imaging of vesicular transport, and phospho-proteomic mapping of MAPK-dependent signaling networks. In addition, single-cell and organoid-based infection models may help resolve cell-type-specific effects and distinguish direct MAPK-dependent mechanisms from parallel pathways such as ER stress responses or lipid metabolic remodeling. Such approaches will be essential to determine whether MAPKs act as primary regulators of CD1d biology or as context-dependent modulators within a broader network governing iNKT cell activation during viral infection.

For certain viruses, the relationship between MAPK signaling and CD1d regulation remains indirect or uncertain, highlighting the heterogeneity of this axis (Table 2). In the case of VZV, CD1d downregulation is well established; however, the underlying mechanism appears to involve altered protein trafficking or transcriptional regulation and is currently considered MAPK-independent or uncertain, as direct evidence linking this process to MAPK signaling is lacking. Similarly, vaccinia virus proteins B1R and H5R inhibit CD1d-mediated antigen presentation, yet their connection to MAPK pathways appears indirect and remains unresolved, with no definitive mechanistic evidence supporting MAPK-dependent regulation. Together, these examples underscore the need to distinguish experimentally validated MAPK-driven mechanisms from virus-specific effects that may operate through alternative or overlapping pathways, rather than assuming a uniform MAPK–CD1d regulatory axis across infections (Table 2).

In addition to MAPK signaling, several non-kinase mechanisms also regulate CD1d functionality, often operating upstream or parallel to MAPK activity. Host lipid metabolism, for instance, is crucial for the proper folding and antigen loading of CD1d molecules. Viruses can reprogram lipid biosynthesis and trafficking pathways, thereby indirectly reducing CD1d stability and surface expression [22]. Likewise, persistent infections often trigger ER stress and activate the unfolded protein response (UPR), which may misroute CD1d or delay its maturation. While MAPKs can influence components of the UPR, these effects are not uniformly kinase-dependent and may vary by cell type and infection stage [62]. Some viruses go further, targeting CD1d for degradation through ubiquitin-proteasome pathways, using viral proteins to recruit host E3 ligases, a strategy that bypasses MAPK signaling entirely [26]. Moreover, endosomal sorting defects can trap CD1d in compartments that prevent antigen presentation. Although MAPKs may regulate parts of this process through cytoskeletal dynamics or Rab GTPases, many of these trafficking decisions are independent of kinase input and governed by vesicle-specific cues [63].

Altogether, the regulation of CD1d antigen presentation during viral infection involves a complex interplay between MAPK pathways and alternative cellular mechanisms. Clarifying this network is essential for determining whether targeting MAPKs alone will be sufficient to restore iNKT cell-mediated immunity, or if broader, combinatorial strategies will be required to reverse viral subversion of this critical immunological axis.

While the idea of a centralized “MAPK–CD1d axis” in viral immune evasion has gained interest, current evidence suggests that this interaction is far more virus-specific and mechanistically diverse than previously assumed. For example, in HSV-1, downregulation of CD1d has been linked to the viral protein ICP47, which interferes with ER-to-Golgi trafficking of antigen-presenting molecules (Figure 3). However, this suppression does not appear to involve classical MAPK pathways like p38 or ERK, as direct kinase involvement remains unconfirmed [26]. In the case of vaccinia virus, the viral proteins B1R and H5R inhibit CD1d-mediated antigen presentation, but it is still unclear whether this effect is mediated via host MAPK modulation or through entirely separate signaling pathways [28].

HCMV and VZV also disrupt CD1d function through distinct mechanisms, such as altering vesicular trafficking or downregulating CD1d at the mRNA level, possibly bypassing MAPK signaling altogether [64]. These differences highlight the need to avoid overly broad generalizations when discussing MAPK’s role in CD1d regulation. Instead, future research should focus on pathogen-specific and cell-type-dependent mechanisms, carefully distinguishing validated MAPK-dependent effects from those that are speculative or context-limited. The idea of restoring CD1d surface expression to enhance iNKT cell-mediated immunity offers a promising therapeutic strategy, particularly in chronic viral infections where this pathway is frequently compromised. However, therapeutic modulation of MAPKs to achieve this goal must be approached with caution. For instance, activating ERK signaling has been proposed as a means to improve CD1d trafficking, but this approach is limited by ERK’s well-established role in promoting cell proliferation and oncogenesis, raising safety concerns in clinical settings [19].

Natural compounds and small molecules like Imiquimod and Probenecid have shown potential to modulate MAPK pathways and stimulate immune responses in vitro [13,65,66], but their specific effects on CD1d expression and iNKT cell activation remain poorly studied in vivo. Moreover, MAPK pathways regulate multiple arms of immunity, including MHC expression, cytokine release, and T cell differentiation. Broad or non-specific MAPK targeting could unintentionally suppress protective immune responses or exacerbate inflammation. Therefore, precision is critical. Therapeutic strategies aimed at rescuing CD1d function via MAPK modulation may need to incorporate targeted delivery systems, such as nanoparticles or tissue-specific promoters, and might benefit from temporal control of drug activity. Combining MAPK-based interventions with immune checkpoint agonists could also help rebalance the immune system without triggering widespread dysregulation. Overall, while the MAPK–CD1d axis holds promise, its therapeutic targeting requires nuanced design and careful validation.

Importantly, the extent to which MAPK signaling directly regulates CD1d trafficking and function varies considerably across viral systems. While some studies provide clear mechanistic evidence linking MAPK activation, particularly p38, to impaired CD1d antigen presentation, as demonstrated in vesicular stomatitis virus models, other associations remain indirect or incompletely defined. For viruses such as vaccinia virus and VZV, CD1d downregulation is well documented; however, the involvement of MAPK pathways is largely inferred or speculative, with limited experimental validation. This heterogeneity underscores the need to distinguish validated MAPK-dependent mechanisms from context-dependent or hypothetical models, and cautions against generalizing a unified MAPK–CD1d axis across diverse viral infections. Future studies should prioritize dissecting these pathways using virus-specific and cell-type-resolved approaches to establish causal links between MAPK signaling and CD1d regulation.

**Table 2:** CD1d–iNKT Axis Modulation through MAPK Signaling in Viral Infections.

Virus	Viral Protein(s)	MAPK Pathway Involved	Mechanism of CD1d Modulation	Ref.
HSV-1	ICP47	Not fully MAPK-dependent	Blocks ER-to-Golgi trafficking of CD1d, limiting surface expression	[26]
Vaccinia Virus	B1R, H5R	Possible MAPK involvement	Inhibits CD1d-mediated antigen presentation	[28]
VZV	Unspecified	Unknown	Downregulates CD1d expression, potentially at transcriptional or trafficking level	[27]
HCMV	US2, US11, Others	Possibly p38/ERK	Alters CD1d trafficking and surface levels; mechanism may include misrouting	[39,49]
SARS-CoV-2	ORF7a, ORF3a	p38 MAPK	Interferes with antigen presentation machinery (primarily MHC-I, possible CD1d overlap)	[43,53,64]
VSV (Vesicular Stomatitis Virus)	Matrix protein	ERK/p38	Suggested to impair CD1d surface localization through MAPK signaling interference	[24,28]
EBV (Epstein–Barr Virus)	LMP1	ERK, p38	Modulates DC function and CD1d-related presentation through MAPK signaling	[36,64]

While emerging studies have begun to elucidate the role of MAPK signaling in CD1d regulation, the current evidence base remains fragmented and warrants careful interpretation. Despite growing interest in the MAPK–CD1d axis, several important limitations constrain current understanding. Much of the available evidence is derived from in vitro or reductionist model systems, which may not fully capture the complexity of tissue-specific immune environments and multicellular interactions present in vivo. In addition, mechanistic insights are often based on pharmacological inhibition or correlative observations, making it difficult to establish direct causal links between specific MAPK pathways and CD1d trafficking or function. Notably, there remains a scarcity of studies directly demonstrating MAPK-dependent regulation of CD1d across diverse viral infections, with robust evidence largely limited to select models, while for others (e.g., vaccinia virus or varicella-zoster virus) the connection remains indirect or unresolved. Furthermore, the contribution of factors such as lipid metabolism, endosomal trafficking, and ER stress responses is frequently not disentangled from MAPK signaling, complicating interpretation. Addressing these gaps will require integrated in vivo models, cell-type-specific analyses, and mechanistic approaches that move beyond association toward causality, thereby refining our understanding of how MAPK signaling shapes CD1d-mediated immunity during viral infection.

## 7. Recalibrating Therapeutic Strategies: Targeting MAPK Pathways with Precision

**Figure 4:**
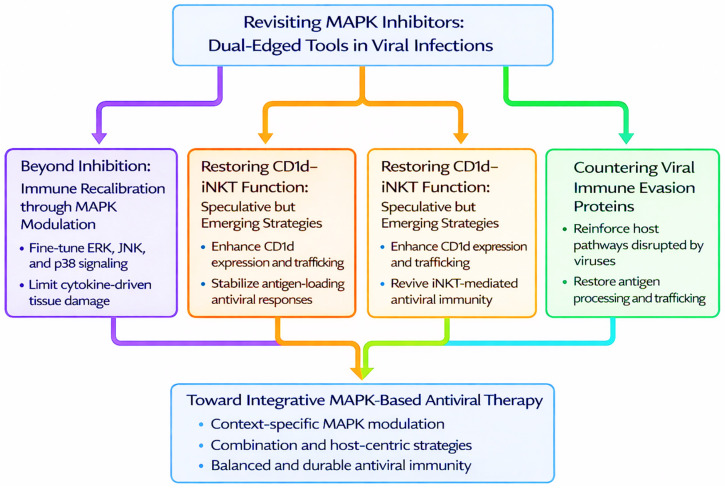
Precision Targeting of MAPK Pathways for Antiviral Immune Recalibration. Strategic framework summarizing the transition from broad MAPK inhibition toward precision-based modulation in viral infections. Conventional MAPK inhibitors are highlighted as dual-edged tools with context-dependent benefits and risks. Emerging approaches emphasize selective tuning of ERK, JNK, and p38 signaling to rebalance immune responses, restore CD1d–iNKT cell function, and reinforce host pathways disrupted by viral immune evasion. These complementary strategies converge toward integrative MAPK-based antiviral therapies designed to preserve antigen presentation, limit immune pathology, and support durable, context-sensitive antiviral immunity.

MAPKs play a central role in viral pathogenesis by shaping immune responses, regulating cytokine production, antigen presentation, and T cell function (Figure 4). While these pathways offer attractive targets for therapeutic intervention, their complex and cell-type-specific roles also pose significant risks. Broad activation or inhibition may disrupt protective immune mechanisms or promote pathological outcomes such as immunosuppression or tumorigenesis. Therefore, future strategies must shift from generalized inhibition toward precision-based approaches that account for timing, cellular context, and viral pathogenesis.

### 7.1. Context-Dependent MAPK Targeting: From Broad Inhibition to Precision Immune Modulation

MAPK-targeted therapies in viral infections exhibit context-dependent efficacy, reflecting the pleiotropic and stage-specific roles of ERK, JNK, and p38 signaling pathways. Among these, p38 inhibitors such as SB203580 and BIRB796 have demonstrated the capacity to reduce pro-inflammatory cytokine production (e.g., IL-6, TNF-α) in lung epithelial and ex vivo SARS-CoV-2 models, typically at micromolar concentrations, with variable preservation of interferon responses [65]. However, their impact on viral replication remains inconsistent, and systemic inhibition carries the risk of impairing antiviral immunity. Similarly, ERK pathway inhibitors can suppress viral replication in influenza and HIV models by disrupting nuclear export and transcriptional processes [16,24], but may concurrently attenuate T cell activation, whereas JNK inhibition can reduce apoptosis while compromising antiviral cytokine signaling [7]. These mixed outcomes underscore the limitations of non-selective MAPK inhibition, which has shown limited translational success due to off-target effects and immune suppression. Importantly, these outcomes reflect the broader challenge that MAPK modulation can produce unintended immunological and cellular consequences. For instance, ERK inhibition may impair cell proliferation, tissue repair, and T cell activation, thereby compromising immune competence. Similarly, the effects of p38 inhibition are highly time-dependent: early suppression may blunt interferon-mediated antiviral defenses, whereas later intervention may help mitigate immunopathology. JNK modulation presents a comparable duality, where reduced apoptosis may preserve tissue integrity but interfere with cytokine signaling required for effective immune responses. These risks highlight the necessity for precision-guided therapeutic strategies that account for timing, cellular context, and disease stage.

In response, therapeutic strategies are increasingly shifting toward a hierarchical framework of precision modulation, including: (i) selective pathway biasing at upstream nodes (e.g., Raf/MEK) to redirect downstream signaling toward beneficial immune outcomes [10]; (ii) temporal modulation, aimed at preserving early MAPK-dependent antiviral responses while attenuating late-stage immunopathology; and (iii) targeted delivery approaches, such as nanoparticle-based systems, to restrict pathway modulation to infected tissues or specific immune cell subsets [17]. Emerging strategies, including MAPK-responsive synthetic circuits in engineered T cells, further highlight the potential for dynamic and signal-dependent immune tuning. Collectively, these approaches reflect a transition from broad pathway inhibition to precision-guided immune recalibration.

Despite these advances, the clinical translation of MAPK-targeted and CD1d-based interventions remains limited and faces several practical constraints. A key challenge lies in the limited availability of robust clinical data, as most current evidence is derived from preclinical models or repurposing studies, restricting conclusions about efficacy and safety in human viral infections. In addition, therapeutic strategies targeting the CD1d–iNKT axis are hindered by delivery and implementation barriers, including inefficient in vivo targeting, instability of lipid antigens, and variability in iNKT cell responsiveness across individuals. Furthermore, the integration of MAPK modulation with immune-based therapies is complicated by heterogeneous patient responses and disease-specific variability, which are not yet adequately captured in existing models. These limitations highlight the need for improved translational frameworks, including clinically relevant infection models, biomarker-guided patient stratification, and optimized delivery systems, to bridge the gap between mechanistic insight and therapeutic application.

A critical determinant of clinical translation is the identification of appropriate disease contexts, patient populations, and intervention windows. In acute viral infections characterized by hyperinflammation, such as severe influenza or SARS-CoV-2, short-term and controlled inhibition of p38 signaling may help limit tissue damage while preserving essential antiviral responses. In contrast, chronic infections including HIV, HBV, and HCV are associated with sustained MAPK dysregulation that contributes to immune exhaustion, impaired antigen presentation, and dysfunctional T cell responses; in these settings, therapeutic strategies may require selective pathway recalibration rather than broad inhibition, with the goal of restoring immune competence. Importantly, timing of intervention is critical: early-phase infection relies on intact MAPK signaling to support interferon-mediated defenses, whereas later stages may offer a window for targeted modulation to reduce immunopathology or reverse immune dysfunction. Furthermore, patient stratification based on inflammatory status, viral burden, and MAPK activity signatures may improve therapeutic precision. The development of cell-type-specific and tissue-targeted delivery systems, including nanoparticle-based platforms, further enhances the feasibility of modulating MAPK pathways while minimizing systemic toxicity and oncogenic risk. Together, these considerations establish a context-aware, precision-guided framework for MAPK-based antiviral therapy.

### 7.2. Restoring CD1d–iNKT Function: Speculative but Emerging Strategies

Enhancing CD1d expression to revive iNKT cell-mediated antiviral responses is an emerging therapeutic concept, particularly relevant to chronic infections where this axis is suppressed [26,28]. ERK activation has been associated with improved CD1d trafficking, but concerns remain due to ERK’s role in promoting uncontrolled cell proliferation [19]. Experimental agents such as CD1d-stabilizing molecules or synthetic lipid antigens show promise in enhancing antigen loading, though they lack validation in vivo. Similarly, MAPK modulators like Imiquimod and Probenecid have demonstrated indirect effects on CD1d and iNKT cells in vitro [65,66,67], but clinical relevance is unclear. Given the limited mechanistic clarity and translational data, the CD1d–MAPK–iNKT axis remains a promising but unproven therapeutic target.

### 7.3. Countering Viral Immune Evasion Proteins: A Longer-Term Vision

Some viruses express proteins that directly disrupt MAPK signaling or interfere with antigen presentation. HSV-1 ICP0, HCMV US2/US11, and SARS-CoV-2 ORF7a/ORF3a are notable examples, targeting components of the immune machinery to evade detection [26,43]. However, their intracellular localization and multifunctional nature make them difficult to target with drugs. An alternative strategy involves reinforcing host pathways affected by these viral proteins. Enhancing CD1d recycling, stabilizing antigen-processing compartments, or bypassing trafficking bottlenecks may restore immune surveillance even in the presence of viral interference. This host-centric approach, while in early stages, may offer a more durable and adaptable solution than direct antiviral targeting.

### 7.4. Toward Integrative MAPK-Based Antiviral Therapy

With increasing understanding of MAPK complexity, therapeutic approaches must evolve to reflect the dynamic roles of ERK, JNK, and p38 across different infection stages and cell types. Systems-level modelling of MAPK signaling, integrating temporal, spatial, and cell-specific data, can help identify intervention points that minimize immune disruption. Combination therapies, such as MAPK inhibitors paired with immune checkpoint agonists, metabolic modulators, or RNA-based antivirals, may improve outcomes by simultaneously targeting viral replication and immune dysfunction. Stratifying patients based on MAPK activity profiles using omics-based tools could help personalize therapy. Ultimately, MAPKs should be viewed not as binary immune regulators, but as tuneable signaling hubs, central to a new generation of precision antiviral therapies.

## 8. Future Directions

Despite decades of research, the roles of MAPK pathways in viral infections remain only partially understood, particularly when it comes to their interplay with non-classical antigen presentation and immune evasion strategies. Future studies must prioritize mechanistic dissection of virus-specific MAPK manipulations using cell-type-resolved models, such as organoids, single-cell phospho-profiling, and spatial transcriptomics. Particular attention should be paid to the MAPK–CD1d–iNKT axis, which remains underexplored in vivo despite its immunological relevance. In parallel, advances in synthetic biology and CRISPR-based editing could enable programmable modulation of MAPK nodes, offering experimental platforms to uncouple protective from pathological signaling outputs. Moreover, the development of biomarkers to assess MAPK pathway activity across infection stages could support patient stratification in clinical trials. Given the context-dependent nature of MAPK functions, therapeutic innovation must shift from uniform inhibition to dynamic, precision-targeted recalibration strategies that account for timing, tissue specificity, and host variability. Ultimately, integrating computational modelling and rational drug design holds promise to transform our ability to harness MAPKs for antiviral immunotherapy.

## 9. Conclusions

MAPK pathways sit at the intersection of viral sensing, immune regulation, and host–pathogen conflict. This review underscores that MAPKs are not merely linear signaling conduits but context-sensitive regulators whose roles vary across viral species, immune compartments, and infection stages. While viruses exploit these pathways to suppress antigen presentation, modulate cytokine profiles, or evade T cell responses, host cells can use the same nodes for antiviral defense. Among the most compelling but underexplored frontiers is the MAPK-mediated regulation of CD1d trafficking and iNKT activation, a non-classical immune axis that many viruses appear to target, yet remains poorly characterized. The therapeutic landscape is evolving from broad inhibition to pathway recalibration, but much remains speculative. To move from concept to clinic, future interventions must embrace the complexity of MAPK biology, balancing immune restoration with oncogenic risk, and tailoring strategies to the unique immunological terrain of each infection. Only through such multifaceted approaches can we unlock the full potential of MAPK-targeted antiviral therapies.

## Data Availability

No new data were created or analyzed in this study.

## Supplementary Materials

The following supporting information can be downloaded at: https://www.mdpi.com/article/10.3390/pathogens15040384/s1, Table S1: Comparative Features of Canonical MAPK Signaling and Viral Modulation Strategies. References [1,4,5,6,9,10,11,12,14,16,18,20,21,26,28,29,32,36,40,41,43,44,46,49,51,52,54,55,56,61,63,66,67] are cited in the Supplementary Text.
