# Supplementary material for: Mitogen-Activated Protein Kinases: Therapeutic Signaling Catalysts in Viral Immune Evasion
Source: Pathogens. 2026 Apr 3;15(4):384. doi: 10.3390/pathogens15040384 (PMC13119128; doi:10.3390/pathogens15040384)
Supplement: Supplementary file 1 [file pathogens-15-00384-s001.zip › pathogens-4221361-supplementary.pdf]

Supplementary Table S1. Comparative Features of Canonical MAPK Signaling and Viral Modulation Strategies

Feature	Canonical MAPK Signaling (Host Cells)	Virus-Mediated MAPK Modulation	Examples / Ref.
**Upstream Activation**	Triggered by physiological stimuli (growth factors, cytokines, stress signals) via receptor tyrosine kinases (RTKs), GPCRs, and PRRs	Activated or suppressed by viral proteins independent of normal receptor engagement	HSV-1, HCMV activate ERK via viral proteins; SARS-CoV-2 modulates MAPKs via accessory proteins [14,32,46]
**Signal Initiation Control**	Highly regulated, ligand-dependent activation with defined thresholds	Often receptor-independent, hijacked directly at MAP3K/MAP2K or scaffold levels	Viral proteins bypass upstream checkpoints [12, 36]
**Temporal Dynamics**	Transient and tightly controlled activation with feedback inhibition	Sustained, oscillatory, or biphasic activation tailored to infection stage	Early transient ERK activation (HCMV); prolonged p38 activation (SARS-CoV-2) [4,32,66]
**Spatial Regulation**	Compartmentalized signaling (cytosol → nucleus) with controlled localization	Mislocalized or redistributed signaling to favor viral replication niches	Viral manipulation of nuclear vs. cytoplasmic MAPK pools [1,10]
**Pathway Specificity**	Coordinated activation of ERK, JNK, and p38 depending on stimulus	Selective activation or suppression of specific MAPK branches	HIV activates ERK in T cells but suppresses p38/JNK in APCs [16, 40]
**Signal Intensity**	Balanced activation to maintain homeostasis	Amplified or dampened signaling to alter immune outcomes	Hyperactivation (cytokine storm) vs. suppression (immune evasion) [41,56]
**Feedback Regulation**	Negative feedback loops (e.g., DUSPs, phosphatases) ensure signal termination	Feedback loops disrupted or co-opted to sustain signaling	Vaccinia virus encodes phosphatases (e.g., VH1) altering MAPK feedback [51,52]
**Crosstalk with Other Pathways**	Integrated with PI3K/Akt, NF-κB, JAK/STAT pathways in a regulated manner	Rewired crosstalk to favor viral survival and replication	MAPK–NF-κB decoupling in viral immune evasion [12,20]
**Downstream Outputs**	Controlled gene expression, proliferation, differentiation, apoptosis, cytokine production	Skewed outputs: immune suppression, altered apoptosis, enhanced viral replication	Reduced antigen presentation, altered cytokine profiles [6,9]

Feature	Canonical MAPK Signaling (Host Cells)	Virus-Mediated MAPK Modulation	Examples / Ref.
**Antigen Presentation**	Supports MHC I/II expression and co-stimulatory signaling under immune activation	Impaired antigen presentation (MHC, CD1d) via MAPK-dependent or independent mechanisms	CD1d downregulation (HSV-1, Vaccinia, VSV) [21,26,28,61]
**T Cell Function**	Promotes balanced effector and memory T cell responses	Drives T cell exhaustion, impaired IL-2 production, PD-1 upregulation	Chronic HIV, HBV, HCV infections [29,54,55]
**Cell Fate Decisions**	Regulates apoptosis, survival, and differentiation in a controlled manner	Either inhibits apoptosis (to prolong infection) or induces it (to aid spread)	HCV (anti-apoptotic), JNK-mediated apoptosis in some infections [5,44]
**Metabolic Integration**	Coordinates glycolysis and mitochondrial function in immune cells	Reprograms host metabolism to favor viral persistence	MAPK-driven metabolic rewiring in chronic infections [43,63]
**Systemic Outcome**	Maintains immune homeostasis and effective pathogen clearance	Promotes immune evasion, persistence, and immunopathology	Cytokine storm vs. immune exhaustion [11,49]
**Therapeutic Implications**	Targeting requires caution due to essential physiological roles	Opportunity for selective, context-dependent modulation	Need for temporal, cell-specific MAPK targeting strategies [18,66,67]
